# Mental Health, Discourse and Stigma

**DOI:** 10.1186/s40359-023-01210-6

**Published:** 2023-06-12

**Authors:** Olga Zayts-Spence, David Edmonds, Zoe Fortune

**Affiliations:** School of English, The University of Hong Kong, Pokfulam, Hong Kong

**Keywords:** Mental health, Discourse, Stigma, Sociolinguistics

## Abstract

In this editorial to the special collection “Mental Health, Discourse and Stigma” we outline the concepts of mental, health, discourse and stigma as they are examined through sociolinguistic lenses. We examine the sociolinguistic approach to mental health and stigma and discuss the different theoretical frameworks and methodological approaches that have been applied in such contexts. Sociolinguistics views mental health and stigma as discursively constructed and constituted, i.e. they are both manifest, negotiated, reinforced or contested in the language that people use. We highlight existing gaps in sociolinguistic research and outline how it could enrich research in psychology and psychiatry and contribute to professional practice. Specifically, sociolinguistics provides well-established methodological tools to research the ‘voices’ of people with a history of mental ill health, their family, carers and mental health professionals in both online and off-line contexts. This is vital to develop targeted interventions and to contribute to de-stigmatization of mental health. To conclude, we highlight the importance of transdisciplinary research that brings together expertise in psychology, psychiatry and sociolinguistics.

## Introduction

This special collection brings together the three broad themes of mental health, discourse and stigma as they are examined through sociolinguistic lenses. We first present what we mean by mental health, discourse and stigma and discuss the interrelationships between these concepts. We then offer a brief overview of existing sociolinguistic research on mental health and stigma and identify continuing areas of under-research that we hope this special collection will contribute to. Finally, we ask the questions of ‘why?’ and ‘so what?’ in relation to sociolinguistic research on mental health and stigma and outline some ways in which this growing area of research could meaningfully contribute to broader professional practice in psychology and psychiatry.

### Defining mental health, mental health stigma and discourse

A range of theoretical frameworks and methodological approaches have been used to investigate mental health and stigma across different disciplines, including sociolinguistics. Historically, the study of mental health has been dominated by psychology and psychiatry which has led to a “psychiatrization” [1] of mental health and illness, approaching the matter from a clinical perspective. In this special collection, we adopt the World Health Organization’s (WHO) encompassing definition of mental health as “a state of mental well-being that enables people to cope with the stresses of life, realize their abilities, learn well and work well, and contribute to their community” [2]. The WHO’s description of mental health acknowledges that “mental health is broader than the lack of a mental disorder”, and encompasses mental disorders, psychosocial disabilities, and “other mental states associated with significant distress, impairment in functioning, or risk of self-harm” [2]. This definition also emphasizes the close interrelationship between mental health and social aspects of life.

Closely intertwined with the concept of mental health is the concept of stigma that has also been widely researched in psychology, psychiatry, as well as sociology. In his seminal essay, sociologist Erving Goffman [3] defines stigma as “an attribute that is deeply discrediting”. Stigmatized individuals are often perceived as being different and “lesser” than “the normals” [3]. Contemporary definitions of mental health stigma largely follow Goffman’s work, highlighting its discrediting attributes and the negative attitudes attached to it [4, 5]. These attitudes also extend to people in the immediate surroundings, such as family [6], and even mental health professionals [7, 8]. This is known as associative or courtesy stigma, i.e. being stigmatized because of a relationship to an individual experiencing a mental health problem [9]. Long describes associative stigma experienced by mental health professionals who are stigmatized because of being “attached to their patients and […] positioned as a less prestigious branch of a broader medical profession” [7]. Shipman and Zayts discuss an extreme case of such associative stigma where a psychiatrist practising in Hong Kong is stigmatized by his own family for working with mentally ill people and for fear of ‘transmission’ of a mental illness [8].

Psychological research on mental health stigma has burgeoned since Goffman’s study. Focusing on the “micro-level social interactions”, this research has examined the causes of stigma, its cognitive dimensions, the consequences and the coping responses [10, 11]. Fewer sociological studies existed [10], but following a much-cited publication by Link and Phelan [12], sociological research has proliferated. Link and Phelan re-defined stigma, surmising it to four processes: labelling differences, stereotyping differences, separating the stigmatized from ‘us’, and discriminating against the stigmatized [12]. By highlighting the processes of discrimination and separation of the stigmatized from ‘us’, Link and Phelan essentially expanded the definition of stigma to the macro-level social processes, such as social inequality, discrimination based on one’s mental health status, and discriminating societal ideologies. These macro-social forms of stigma are known as structural stigma [11]. Workplace settings are one example when employers are hesitant to hire or promote people with a history of mental health problems, although these discriminatory attitudes are typically subtle and indirect. Sociological research has largely focused on how different types of stigma contribute to inequalities and impact social relationships between different groups [10].

Sociolinguistics studies the interrelationship between language and society [13] of which discourse is a central sociolinguistic concept. It is equally as nebulous and multifaceted as the concepts of mental health and stigma. Discourse may refer to:

- A stretch of language above a clause or a sentence level [14].
- Language used by speakers to covey and negotiate certain meanings and achieve particular purposes [15].
- Language used to represent various social practices, social actors and ideologies [16, 17].

Relating these different interpretations of discourse to mental health contexts, in its micro-analytic understanding discourse may refer, for example, to negative stereotypical attributes, such as ‘mad’, ‘crazy’, and ‘insane’. Culturally, there exist striking differences in how mental health disorders are described, although negative connotations typically prevail. For example, the term 精神分裂症 in Chinese (jīng-shén-fēn liè zhènɡ, schizophrenia) has a literal translation of ‘the split-mind disease’. This term is heavily stigmatizing. Substantial efforts have been made by mental health professionals in Hong Kong to introduce less stigmatizing terms, such as 思覺失調 (sī jué shī tiáo, psychosis) that translates as ‘thought and perceptual dysregulation’ [18].

In relation to discourse as ‘language-in-use’, the following example from an interview with a psychiatrist illustrates how the diagnosis of a ‘schizophrenic patient’ is used to account for the tragic event of mass killing that has marked, in this psychiatrist’s words, “a milestone” in the development of psychiatric services in Hong Kong. It is an objectivized, ‘clinical’ account of a mental health disorder and its impact on one’s behaviour. It also highlights a wider societal impact in response to the recounted incident.

#### Example 1

*Psy – psychiatrist; I – interviewer*.

6. Psy: Yes. The most well known in Hong Kong is this 1982 tragedy […] Schizophrenic patient, young man killed his mother and sister at home [.] and then went down with two knives and entered a kindergarten and killed four more.
11. I: Children?
12. Psy: Four kids and wounded forty something. […] This incident created, it may be regarded as a milestone for the development of the service, because after this incident all the society turned attention to mental health, mental patients.

The last approach to discourse foregrounds how mental health is constructed through social practices. Mental health and mental health stigma are “socially and discursively constituted” [19] with a bidirectional relationship between discourse and social practices. Crudely speaking, discourse is the ‘mirror’ through which social practices and ideologies become evident. For example, different linguistic choices, such as the use of derogative, direct or figurative language to talk about mental health, reflect the dominant societal practice and ideologies. In the reverse, ways of communicating about mental health impact social practices and ideologies. One example could be media portrayals of mental health as both a reflection of prevalent societal ideologies and ways to impact them. These different conceptualizations of discourse have been employed in sociolinguistic studies of mental health and mental health stigma.

### Sociolinguistic research on mental health and mental health stigma

In this special collection, we use the term sociolinguistics broadly to include linguistic approaches and methodologies as diverse as corpus linguistics, different types of discourse analysis (e.g. thematic and critical), conversation analysis, narrative inquiry, to name just a few. While these approaches conceptualise discourse differently, most sociolinguistic studies on mental health and mental health stigma take a social constructivist view, viewing language as a means of constructing social reality. Sociolinguistic studies include quantitative, qualitative and mixed methods studies. They examine diverse discourse data, from interactions in clinical contexts, to online interactions between members of mental health support groups, to large media corpora. Each of these different types of data provides insights into different aspects of mental health and has both strengths and limitations.

Arguably, one of the most potent sociolinguistic approaches to mental health research to date has been corpus linguistics [19–23]. Corpus linguistics refers to methods that use computerized tools (e.g. Wordsmith, Sketch Engine) to analyse large collections of data (corpora). While the corpus size could be substantial, the use of tools allows consistent and fairly easy identification of patterns in the data [1]. Another common sociolinguistic approach is critical discourse analysis (CDA) [24, 25]. For example, Price uses corpus linguistics and CDA to interrogate news reports on mental health in the UK from 1984 to 2014 [26]. Substantial corpus data delve into media’s portrayals of mental health, and how mental health stigma is created and perpetuated by media.

Notably, sociolinguistic research often includes the ‘voices’ of under-represented, vulnerable or under-researched demographic groups. For example, Galinsky’s and colleagues’ research focuses on discourses surrounding male depression and suicide [27–29]. Societal ideologies around men as strong and powerful often stop men from seeking help and opening up about their mental health struggles. Sociolinguistic research has much to contribute to elucidating these dominant ideologies and support organizations targeting men’﻿s mental health (e.g. Mind UK or Manup). It could also contribute to understanding groups ‘associated’ with people with a history of mental ill health. For example, Ziółkowskaa and Galasiński, examine the narratives of children of fathers who died by suicide and how they deal with both bereavement of their deceased parent and the stigma attached to death by suicide [29].

These are just a few examples of previous and ongoing sociolinguistic research, and in this special collection we welcome contributions that apply different theoretical frameworks and methodological approaches in sociolinguistics.

### The ‘why?’ and the ‘so what?’ of sociolinguistic research on mental health and mental health stigma

This brief overview of sociolinguistic research points to some of the possible applications of research to professional practice. Sociolinguistic research focuses on discourses of mental health. These discourses are powerful, they are the means to talk about mental health, the locale where mental health issues are manifest, the means to seek and offer help, and the ways to offer education and develop interventions. They are also the means to challenge and contest negative ideologies. De-stigmatisation of mental health can be achieved through structural changes (e.g. offering equal employment opportunities) but most, if not all, social activities and practices are mediated through language. Therefore, sociolinguistic research continues to make a strong contribution to mental health de-stigmatization, research and practice.

There is an increasing emphasis in psychology and psychiatry on participatory research, including with vulnerable demographic groups [30]. As this editorial emphasizes, a strength of current sociolinguistic research is that it investigates the ‘voices’ of different groups of people affected by mental ill health. Established sociolinguistic approaches (e.g. narrative inquiry, rhetorical discourse analysis) provide tools to examine different types of accounts for the social actions that people perform, why and when people give accounts, and the language that they use when they do it. Investigating these data is important to develop targeted interventions for different groups of people affected by mental ill health.

Sociolinguistic research also ‘weaves together’ the micro-interactions with other contexts, the meso (e.g. institutional) and the macro (societal), bringing personal and the social aspects of mental health together to provide a more holistic picture.

Our brief overview has identified research gaps. There remains a paucity of empirical sociolinguistic research that uses real-life interactional data in face-to-face communicative encounters in mental health contexts, for example, in counselling or psychotherapy encounters. This may be partly due to ethical considerations of access and the use of sensitive data (for exceptions, see, for example, Lavie and Nakash) [31]. Examining these types of data allows exploring in detail how social actions are accomplished *in situ*, that is, in real time during an interaction, for example, how diagnosis or possible treatment negotiations are accomplished. There is also limited research on inter-professional communication in mental health contexts which could examine the linguistic repertoires of professional practices, professional ethos, and how diagnoses or treatment recommendations are negotiated inter-professionally, among other issues. Research cited in this editorial mostly comes from Anglophone contexts. Research from ‘global peripheries’ [32] remains scarce. While there are a few exceptions, more research from other geographical contexts is called for [33].

To conclude, there are ample opportunities for trans-disciplinary research that brings together expertise in psychology, psychiatry and sociolinguistics. While discursive psychology, for example, has long been concerned with investigating issues pertaining to psychology through language, sociolinguistics offers more versatile and nuanced ways of doing it by offering a range of different approaches and methodologies. In this special collection we welcome contributions that demonstrate the value of sociolinguistic research and how it could enhance existing research and practice in psychology and psychiatry. We invite contributions that draw on diverse empirical data from different clinical and non-clinical contexts and that focus on different mental health conditions. We also welcome authors working in diverse sociocultural contexts whose work could advance our understanding of the cultural aspects present in discourses of mental health and stigma. It is through such trans-disciplinary effort that we can challenge the existing social practices and ideologies of mental health and ultimately contribute to addressing some long-standing societal issues of discrimination and stigmatization of people with mental health issues.

## Data Availability

Not applicable.
